# Object-Aware Computational Integral Imaging for Improved Object Depth Estimation and Stereo Matching Training

**DOI:** 10.3390/s26134149

**Published:** 2026-07-01

**Authors:** Daniel Vais, Yitzhak Yitzhaky

**Affiliations:** Department of Electro-Optics and Photonics Engineering, School of Electrical and Computer Engineering, Ben-Gurion University of the Negev, Beer Sheva 84105, Israel; vaisdani@post.bgu.ac.il

**Keywords:** depth estimation, disparity, stereo matching, computational integral imaging (CII), object detection, segmentation, ROI

## Abstract

Depth estimation is an active area of research in computer vision. When restricted to passive imaging, it is frequently approached as a stereo matching problem. Due to significant advancements in deep learning, stereo matching models have seen substantial development. Most stereo matching models utilize supervised learning, relying on disparity maps as ground truth, typically obtained via active imaging systems. In this work, we introduce a passive multi-view framework for generating ground-truth depth data for stereo matching models using Computational Integral Imaging (CII). Using CII, we extract object depths from multi-view images obtained via a passive camera array and use these depths as training targets, eliminating the need for active depth acquisition. To improve the robustness and accuracy of CII-based depth extraction, we propose an object-aware formulation that incorporates pretrained object segmentation into the depth extraction process. This enables more reliable depth estimation for objects with complex appearances and challenging scene contexts such as occlusions. Furthermore, we exploit the camera array as a multi-stereo acquisition system, generating diverse stereo pairs with varying baselines and viewing orientations. The resulting training data expose stereo matching models to a broader range of geometric configurations than conventional stereo datasets. Our results demonstrate that this approach enhances the generalization capabilities of stereo matching models.

## 1. Introduction

Depth estimation is an important problem in many fields, including autonomous driving [1], robotics [2], medical imaging [3], and general 3D scene understanding. The process is used to determine the distance between objects in a scene and the camera. Accurate depth estimation allows machines to go beyond simple 2D image capture and gain a meaningful 3D understanding of their surroundings.

A widely used approach for depth estimation is stereo matching [4,5]. In stereo systems, two cameras capture the same scene from slightly different viewpoints. By finding correspondences between these images, the system can estimate depth using either classical geometry-based formulas or modern learning-based methods. With the rapid progress of deep learning, learning-based stereo matching has become a highly active area of research.

Integral imaging is a passive imaging method. It captures multiple views of a scene, known as elemental images, which together preserve both spatial and angular information of light. This allows the reconstruction of a 3D representation of the scene. Computational Integral Imaging (CII) performs this reconstruction digitally. CII uses the multi-view input to recover 3D depth information through computation rather than optical projection. As a fully passive method, CII is flexible and cost-effective, making it suitable for scenarios where active sensors such as Light Detection and Ranging (LiDAR) [6] or structured light [7] are less practical. Furthermore, using the same camera system for obtaining both depth data and spatial object appearance simplifies the association of depth data with object Regions of Interest (ROIs), compared to systems combining different imaging modalities, such as LiDAR and RGB cameras [8,9,10,11].

Generalization is a major challenge in stereo matching, as it is in machine learning in general. A model that performs well on one dataset may fail when applied to new data, especially since many stereo datasets are captured using a fixed pair of cameras with a constant baseline. This makes the model dependent on a specific camera setup. Multi-view data helps address this problem by providing a richer set of viewpoints of the same scene. Unlike traditional stereo, which usually relies on a horizontally aligned image pair, a multi-view system allows the construction of diverse image pair types, including horizontal, vertical, and diagonal combinations. In addition, multi-view data provides different baseline distances between cameras, which changes the disparity and scene geometry. Training with stereo pairs generated from diverse alignments and baselines exposes the model to varied geometric conditions, improving its robustness and ability to generalize to different camera setups.

### 1.1. Related Work

Recent advancements in CII depth-based image analysis include the work of [12], which introduced a deep learning integral imaging system that reconstructs 3D images while excluding the out-of-focus areas that appear in the CII reconstructed depth plane. Objects in 2D images are detected and segmented using a pretrained Mask-RCNN algorithm, which are then used for 3D image reconstruction. The method proposed in [13] utilizes a pretrained Mask-RCNN algorithm to identify ROIs (bounding boxes) of multiple objects within a 2D image. These ROIs are then passed through a gradient-based depth extraction using CII data [14,15], to provide depth estimation for each detected object.

Stereo matching is a widely studied problem, including methods that are only geometry-based, deep learning methods, and hybrid methods that combine geometry-based methods with learning algorithms. Classical algorithms were initially based on local window-based comparisons. Block Matching (BM) computes similarity between fixed-sized windows in the left and right images using evaluation metrics such as Mean Absolute Difference (MAD) or Mean Squared Error (MSE). Based on the window-based methods, research introduced improvements such as adaptive support windows [16], which propose support-weights of the pixels in the window based on color similarity to reduce image ambiguity. The authors of [17] proposed using transformations such as rank and census transforms as the basis for correlation, as they tolerate outliers better. Another group of methods consists of global optimization techniques, which look at the problem as an energy minimization problem. Belief Propagation [18] and graph cuts [19] both model the stereo matching problem as a Markov Field optimization problem. Dynamic programming [20] models the stereo matching problem as a search problem. One of the widely used classical methods is Semi-Global Matching [21] (SGM), which approximates 2D global optimization by aggregating costs along multiple paths.

Stereo matching models are typically trained on standard datasets such as Middlebury [22], KITTI [6], and the synthetic Scene Flow dataset [23]. Some deep learning approaches have replaced manual similarity metrics with neural networks. For example, MC-CNN [24] employed a Siamese CNN to compute patch similarity costs, which are then integrated into a traditional stereo pipeline. Next, end-to-end stereo matching models were proposed. PSMNet [25] constructs a cost volume and aggregates context using spatial pyramid pooling and 3D convolutions. GWCNet [26] refines cost volume construction using groupwise correlation. For computational benefits, AANet [27] replaces the costly cost aggregation of 3D convolutions with sparse cost aggregation over selected points. Other contributions include GANet [28], which introduces guided aggregation layers to better capture local details, and RAFT-Stereo [29], which is an iterative refinement approach that updates disparities through recurrent modules. Hybrid approaches consist of methods that combine stereo matching learning algorithms with classical non-learning methods. In addition to MC-CNN [24], SGM-Net [30] introduces a learned penalty method for the classical SGM. Studies have also been conducted on combining structure-from-motion 3D data and multi-view stereo to obtain 3D reconstruction in hyperspectral images [31,32,33].

Guided stereo matching is a method that incorporates depth hints from external sensors, such as LiDAR, into the stereo matching training process. This method uses a LiDAR branch that provides sparse depth information in addition to the dense stereo information branch [34]. Depth maps are widely used as ground-truth data for stereo matching model training. Depth data that is obtained using active imaging may have some drawbacks in certain scenarios. Therefore, in addition to depth maps that are generated using active imaging methods, many stereo matching models are trained on synthetic data as well [35].

### 1.2. Proposed Contribution

In this work, we propose a novel method that performs both ground-truth object depth formation and learning-based stereo matching using a passive camera array [36].

Using a multi-view image array, a CII-based method is able to extract the depth of objects detected in the scene. Similarly to [13], we applied 2D object detection to produce ROIs of the objects in the scene and then performed CII only within these ROIs to determine the depth of each object detected in the scene. However, in this work, we improved this method by enhancing the spatial specificity by implementing object-specific masks as ROIs instead of the bounding boxes used in [11]. This improves the objects’ spatial localization, thus improving CII-based depth estimation, as it does not consider any background that appears within the bounding box of the objects and uses only the real locations of the objects. This masking increases the depth extraction robustness for complicated object appearances and when objects are adjacent to each other in the image.

Next, we used the depths found by the improved masked CII-based method as the ground-truth data to train the stereo matching model. This workflow enables stereo matching models to be trained with only passive imaging inputs, without requiring active imaging to produce ground-truth data [25,26,27,28]. This enables stereo matching models to be trained in scenarios where active imaging is not applicable or less effective, such as live bio-imaging [37] and sunny outdoor scenarios [38]. Unlike active systems, cameras can capture complete images in milliseconds, making them more compatible for use as mobile sensors or for operation in dynamic environments. In addition, cameras are usually more efficient in terms of cost, size, mass, and power requirements [39]. The image array data produced here was further employed as diverse multi-stereo imaging data with different baseline distances and orientations, increasing the generalization in the stereo matching model training.

To summarize, the key innovations of this work are (1) replacing bounding-box ROIs with object masks for more accurate CII-based object depth extraction (Section 3); (2) using the resulting CII-derived object depths as same-modality supervisory depth labels for stereo matching model training, with geometrically diverse stereo pairs for better generalization (Section 4).

***The remainder of the paper is as follows***: In Section 2, Computational Integral Imaging (CII) is described, along with the approach for assessing the depths of objects in the scene. Section 3 presents the proposed improved method for object depth localization using CII with object masks. Section 4 presents the proposed stereo matching model training method using the extracted CII-based depth data. Experimental results and conclusions are presented in Section 5 and Section 6, respectively.

## 2. Object Depth Assessment Using Computational Integral Imaging (CII)

Integral imaging [40] is a method of capturing a scene in 3D using data from multiple views. Integral imaging applies passive imaging, and it can operate in outdoor scenes and under incoherent or ambient light for important applications. With this approach, multiple cameras, arranged in a 2D array, record 2D images of the same scene, each from a slightly different angle. Each captured image is referred to as an elemental image (EI). A 3D image of the scene is reconstructed from these elemental images.

The 3D reconstruction can be achieved optically or computationally. Optical Integral Imaging systems use a lenslet array or an array of cameras to sample the direction and brightness of rays coming from a 3D object. These rays are then recorded using a 2D image sensor. The synthesized image that is reconstructed using CII is of better quality than that of images reconstructed using Optical Integral Imaging [41].

**Figure 1:**
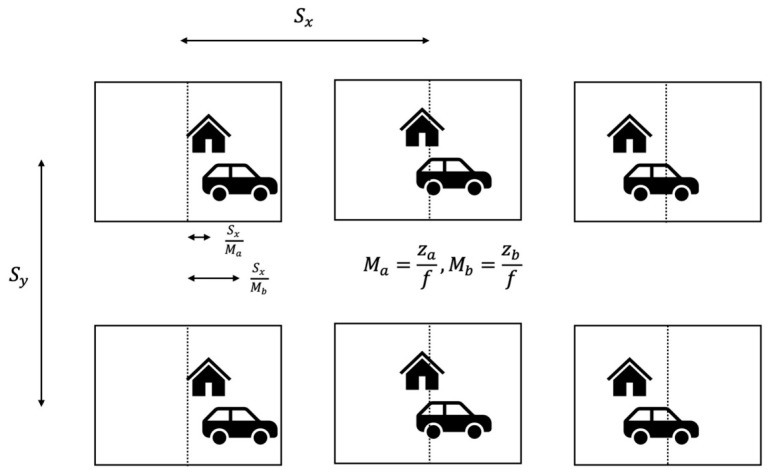
An illustration of six EIs obtained from slightly different angles, with two objects in the scene at depths $z_{a},z_{b}$, Also shown are the imaging setup parameters used in Equation (1).

**Figure 2:**
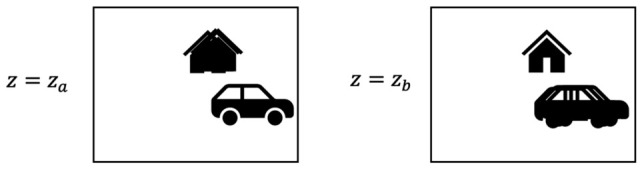
An illustration of two depth planes produced by CII (Equation (1)), one for each object depth. The left image shows the depth plane computed at the car’s depth $z_{a}$, where the car object appears sharp while the house object appears blurred. The right image shows the depth plane computed at the house’s depth $z_{b}$, where the house object is sharp while the car is blurred.

A stack of 2D depth planes is reconstructed, given the recorded image array. Depth planes for a given number of depths are calculated. The 2D reconstructed depth plane for depth $z_{0}$ is given by [13,14]:

(1) $$f\left(x,y,z_{0}\right)=\frac{1}{KL}\sum_{k=0}^{K-1}\sum_{l=0}^{L-1}g_{kl}\left(x+\left(\frac{1}{M_{z_{0}}}\right)S_{x}k,y+\left(\frac{1}{M_{z_{0}}}\right)S_{y}l\right)$$

where ${\;g}_{kl}$ is EI with k,l indices, K×L denotes the number of Eis in the camera array, and$\;M_{z_{0}}$ is the magnification factor that depends on $z_{0}$ and the focal distance *f* (see Figure 1). The coordinates that define the camera’s array plane are x and y, and $S_{x}\;\mathrm{and}\;S_{y}$ are the distances between cameras in the camera array in the x,y plane. In this operation, objects located in the depth of the calculated plane $z_{0}$ will appear sharp in that plane, while objects out of this depth will not be imaged into the same location, and hence, will appear multiple and blurry. An illustration of this process is shown in Figure 1 and Figure 2. Figure 1 illustrates six elemental images (EIs) of two objects in different depths, representing slightly different viewing angles. Figure 2 illustrates two calculated depth planes (Equation (1)), one for each object depth.

Given the sharpness characteristics of the reconstructed depth planes in relation to the object depths, the object depths in the scene are extracted based on a sharpness measure across the depth planes [14,15]. At the depth where the object appears sharpest, the average gradient of the reconstructed image will be the highest, due to a higher gradient value at sharp regions of the image. The Average Gradient Magnitude of the Reconstructed plane (AGMR) at depth $z_{0}$ is given by [13,14,15]:

(2) $$AGMR\left(z_{0}\right)=\frac{1}{N_{x}N_{y}}\sum_{y}\sum_{x}\left|\nabla f\left(x,y,z_{0}\right)\right|$$

where $N_{x}\;\mathrm{and}\;N_{y}$ are the number of pixels in the x and y directions, and ∇ is the gradient operator. Since focused sharp regions in the image are characterized by high gradient values, at each region, the reconstructed plane with the highest AGMR across other depth planes signifies the depth location of the object in that region [14]. In [13], this property was used to extract the depths of detected objects in the scene. Objects in the image of the scene were detected via deep learning, and the AGMR was calculated only within the bounding boxes of each detected object across all reconstructed depth planes, thus producing estimated depth locations of all detected objects.

The depth resolution is defined as the change in depth Δz that corresponds to a change in one pixel in the image. This ability depends on the camera parameters. Therefore, the depth resolution is given by [42]:

(3) $$\Delta z_{min}=\mu \cdot \frac{z^{2}}{p_{max}\cdot f-\mu \cdot z}$$

where z is the object distance (depth), μ is the pixel size, f is the distance between the lens and the sensor, and $p_{max}$ is the maximum parallax.

## 3. Improved Object Depth Localization Using CII via Masks

In this section, we present an improved method for 3D object localization using masks with CII-based depth extraction, instead of using only bounding boxes. In [13], Equation (1) was applied within the bounding box region of each detected object across the reconstructed depth planes. The depth location of each object is defined as the peak of the AGMR curve along the depth axis (i.e., across the depth planes).

However, although bounding boxes provide good localization of objects in a scene, they still include some background regions within them. In fact, in most cases, except for rectangular objects within the horizontal and vertical axes, this ROI does not include only the object itself. Background regions within the bounding box appear blurry because they correspond to objects not located in the depth of the reconstructed plane of that object’s location. Using object masks instead of bounding boxes enables us to use only the pixels of the object itself, excluding any backgrounds.

**Figure 3:**
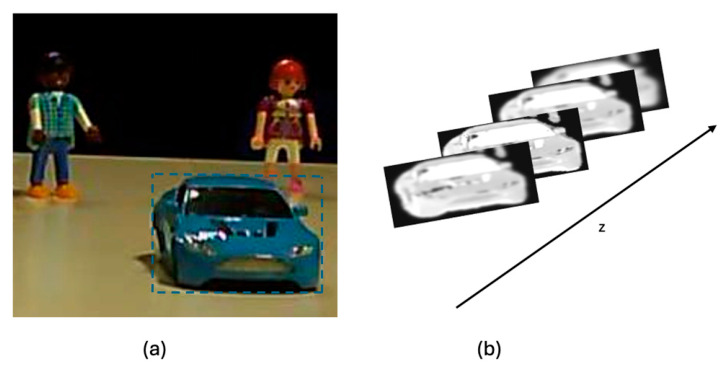
(**a**) An elemental image with a bounding box around a detected car. (**b**) An illustration of four detected object’s ROIs across four reconstructed depth planes (as obtained in Equation (1)). The sharpest reconstructed plane (second from the bottom) corresponds to the object’s depth location, while planes farther from the object’s true depth location appear blurrier.

In this study, we first performed image segmentation using the SAM2 method [43], and then Equation (1) was applied only to the mask pixels across the reconstructed depth planes. Figure 3a presents an image with a bounding box around a detected car object, and Figure 3b illustrates a tube of object masks across four reconstructed planes.

In the next step, we adjust Equation (2) to consider only the masked object area by summing the gradient magnitude only over the object’s pixel locations, forming a more accurate sharpness measure in the reconstructed depth plane of the object at each depth *z* and then determine the depth of the explored object, as follows:

(4) $$AGMR_{O_{i}}\left(z\right)=\frac{1}{N_{x}N_{y}}\sum_{y\in mask_{O_{i}}}\sum_{x\in mask_{O_{i}}}\left|\nabla f\left(x,y,z\right)\right|,$$

where $O_{i}$ represents the *i*-th object detected in the scene, $N_{x}\;\mathrm{and}\;N_{y}$ are the number of pixels in the x and y directions of the detected mask $mask_{O_{i}}$ of object $O_{i}$. This summation that is applied over the mask region differs from that in [13], where it was applied over the whole detected bounding box of the object, which may include non-object and background regions. The region of summation can be slightly larger than the mask to better include gradients in the object boundary region.

The depth location of each object $z_{o_{i}}$ will then be the depth index of the sharpest object’s reconstructed depth plane:

(5) $$z_{O_{i}}=\underset{z}{argmax}\;AGMR_{O_{i}}\left(z\right)$$

As the calculated depth planes (Equation (1)) are farther from the true object’s depth, the object goes out of focus, and its edges become blurrier. This blurriness will always enlarge the object’s image size and make its contours more rounded. Since the mask corresponds to the size of the focused object, in the object’s blurry versions, it will cover the object, but without the fading intensity at the margins induced by the blurriness (as illustrated in Figure 2). Therefore, in each depth plane, the mask will cover the surface of the object (excluding the fading margins), and as the calculated depth plane is farther from the correct depth value, the gradients at this surface will decrease, thus reducing the AGMR sharpness measure.

**Figure 4:**
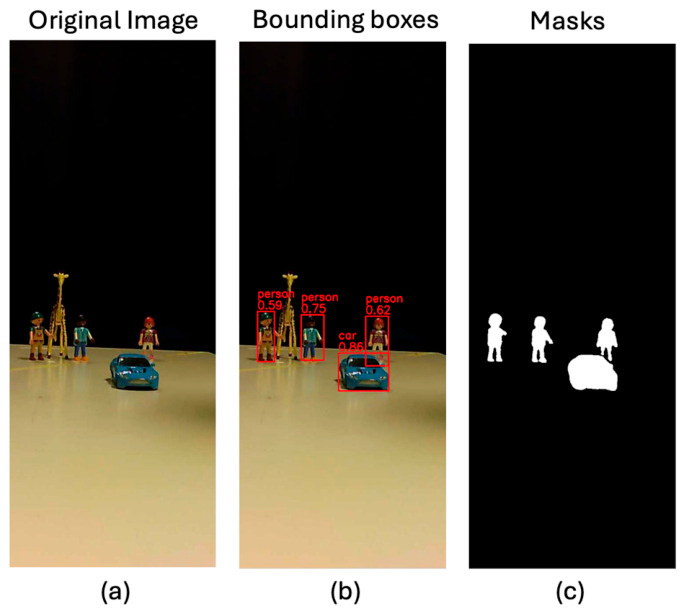
(**a**) An elemental image taken by one of the cameras in the camera array. (**b**) Detected objects with bounding boxes using YOLOv9 [44]. (**c**) Object masks obtained using the SAM2 segmentation model [43].

**Figure 5:**
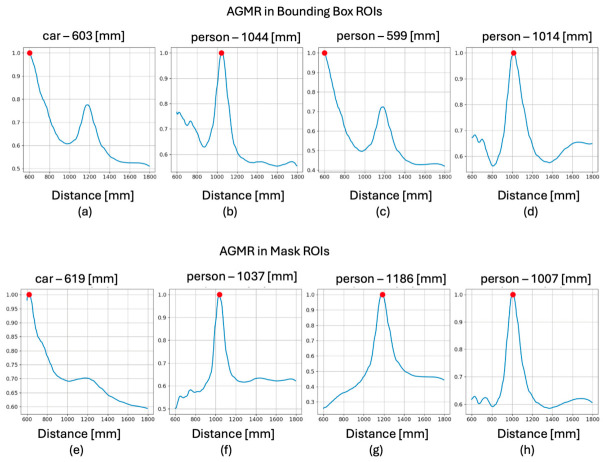
(**a**–**d**) AGMR curves and depth estimations (the red dots indicate the maximum of each curve) obtained using bounding boxes [13] for four objects in the scene. The horizontal axis is the distance from the camera, and the extracted object depth is the distance at the maximum AGMR location. (**e**–**h**) The same as the top row but using object masks instead of bounding boxes. The use of masks produced more accurate object depth positions.

A comparative example of applying both the improved and the previous depth extraction method versions to a real scene is presented in Figure 4 and Figure 5. Figure 4a shows an example of a scene with multiple objects, Figure 4b shows the objects’ detections with their produced bounding boxes using YOLOv9 [44], and Figure 4c presents the objects’ masks obtained via SAM2 [43].

Figure 5 shows the AGMR curves and depth estimations (the distance value at the maximum in each curve) for detected objects in a scene using bounding boxes and using masks.

Figure 5 shows better depth extraction results when using masks compared to bounding boxes. These improvements occurred mainly when the bounding boxes of different objects at different depths overlapped, or more generally, when a background appeared within the bounding box of an object. Such a case can be seen in Figure 4b, where the two right bounding boxes of the car and the person are overlapping. The AGMR curves of these objects using the bounding boxes are shown in Figure 5a,c. These two objects were assigned similar detected depths of 603 mm and 599 mm, respectively, even though in the real scene, the person is significantly farther away (about 1.2 m). The true depth corresponds to the second lower peak. Using masks instead of bounding boxes fixed this error, as can be seen in Figure 5g, where the person object was detected at 1186 mm. For the car case, while the highest peak using the bounding box (Figure 5a) was at the correct depth location of the car, a peak due to the partially overlapping person is also clearly observed. Similarly, using a mask instead of a bounding box (Figure 5e) suppressed this unrelated peak, making the correct peak detection more robust.

**Figure 6:**
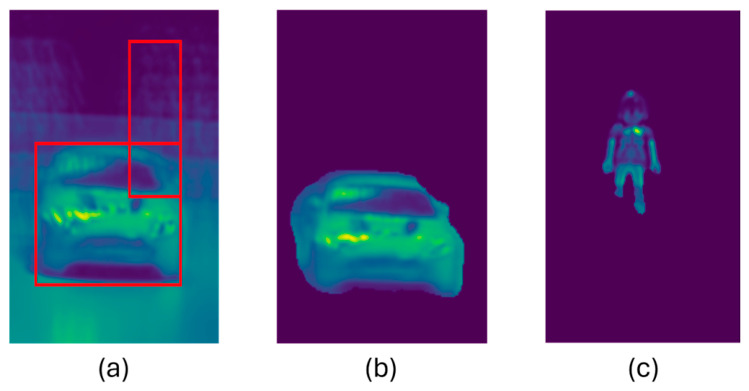
A demonstration of the segmentation effect. (**a**) A reconstructed depth plane region at depth z=599 mm, with bounding box areas of the car and the person. (**b**) The car’s segmented mask region at the reconstructed depth plane at depth z=619 mm. (**c**) The person’s segmented mask region at the reconstructed depth plane region at depth z=1186 mm.

Figure 6 demonstrates how the use of masks is beneficial in scenarios where two objects have overlapping bounding boxes (obtained by YOLOv9 here), as seen in the original image in Figure 4b. Figure 6a shows the reconstructed depth plane at depth z=599 mm when bounding boxes were used. It is clearly seen that due to the overlapping boxes, a part of the car was included in a significant part of the smaller person’s ROI for AGMR calculation at that depth. This resulted in a wrong person depth output (Figure 5c) because the AGMR within the intersecting region was larger than the AGMR at the correct person’s distance. It wrongly detected the person’s depth (599 mm) as very close to the true car’s depth (603 mm—see Figure 6a), which deviates significantly from the actual setup. Figure 6b shows the car’s segmented mask region at the reconstructed depth plane at depth z=619 mm, and Figure 6c shows the person’s segmented mask region at the reconstructed depth plane at depth z=1186 mm. It can be seen that both reconstructions provide an accurate representation of the sharp object within the segmented mask region of the reconstructed depth plane.

## 4. Proposed Method for Stereo Matching Model Training

Depth estimation methods may benefit from additional information, such as object locations and boundaries. Incorporating detection and segmentation into depth estimation pipelines may, in relevant cases, focus the method on meaningful areas while ignoring irrelevant background details.

**Figure 7:**
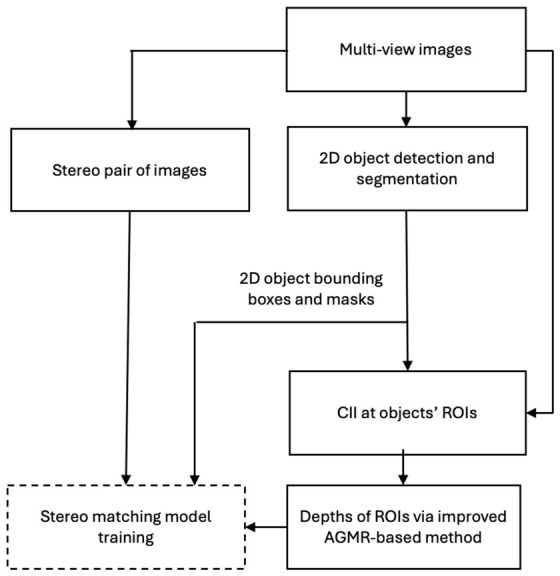
The overall workflow of the proposed method for stereo matching model training. The dashed box is detailed in Figure 8.

We present a method for training stereo matching algorithms using ground-truth data generated by the improved CII-based method presented in Section 3. This non-learning algorithm uses multi-view images from a passive imaging system, which are utilized as a diverse collection of stereo pairs with varying baselines and orientations. The proposed workflow comprises four main stages: multi-view data acquisition, 2D object detection and segmentation, depth reconstruction, and stereo matching training. The overall process is illustrated in Figure 7.

We began by generating the ground-truth data to train the stereo model. A camera array captured multi-view images of the scene, which were used as input for the CII-based algorithm and as input for the stereo matching model, enabling the stereo matching model to be trained on stereo images with varying baselines. Then, 2D object detection and segmentation were performed, applying pretrained SAM2 [43] and YOLOv9 [44] models to one of the multi-view elemental images. YOLOv9 was used to generate object bounding boxes, which were used to ensure the localization of object masks generated using SAM2. Then, a CII-based AGMR was calculated over each object’s mask region (Equation (4)) to generate the depth information of each detected ROI in the scene (Equation (5)). The extracted ROIs’ depths were then used to train the stereo matching model. Finally, we used stereo matching models to generate disparity maps. Combined with the previously generated ROIs and masks, we calculated the depth at the object’s location from the disparity map using [24]:

(6) $$\;z=\frac{f\cdot B}{d}\;\;\;\;$$

where *z* is the depth, *f* is the focal length, *B* is the baseline, and *d* is the disparity. In this way, the stereo matching model was trained in a supervised manner using the CII-based extracted depth as the ground truth.

**Figure 8:**
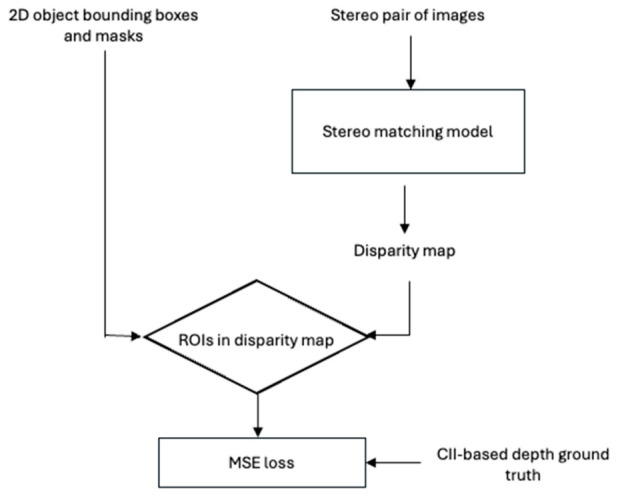
A detailed diagram of the stereo matching training block (in Figure 7) using depth ground truth obtained by the CII-based method.

Figure 8 provides a detailed scheme explaining the stereo matching model training process (the dashed box in Figure 7). Instead of comparing predicted disparity maps to ground-truth disparity maps [25,26,27,28], we located the ROIs within the predicted disparity map using the object masks extracted in a previous step, and then calculated the predicted depths using Equation (6) within these areas. Then, we compared the depths obtained from the predicted disparity map with the ground-truth depths obtained by the CII-based algorithm via an MSE cost function in the stereo matching model training process.

## 5. Experimental Method

**Figure 9:**
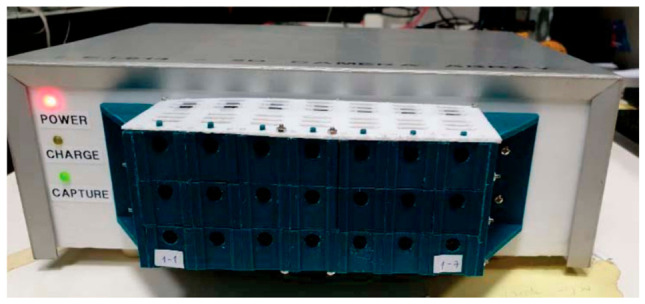
The camera array, containing 3-by-7 mini cameras, spaced 21.1 mm apart in both directions.

**Figure 10:**
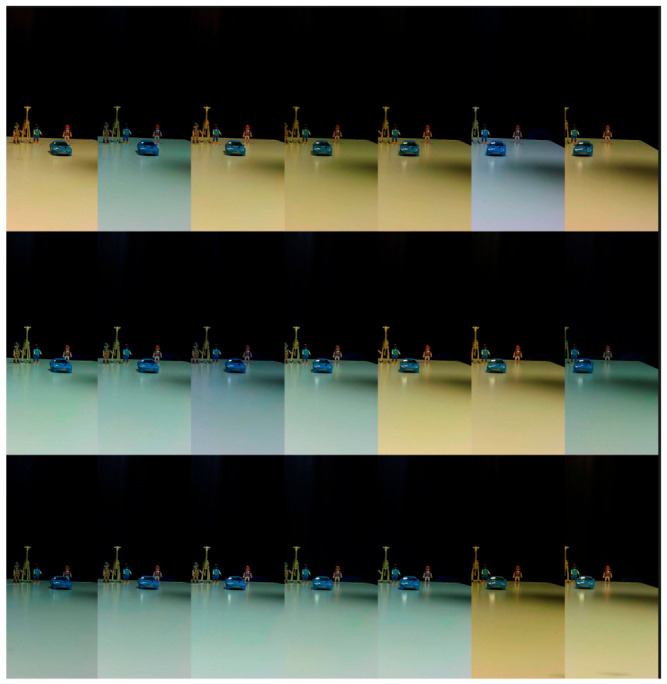
An example of the camera array output (3 × 7 elemental images), capturing a scene of toy objects from slightly different viewpoints. The upper-left elemental image, enlarged, is shown in Figure 4a.

To obtain multi-view images, we used a camera setup developed in our lab [36], which simultaneously captured an array of images with slightly different angles. The camera setup included 21 small cameras, SQ11 mini-HD cameras (Shenzhen Litian Century Security Technology Co., Ltd., Shenzhen, China), arranged in a 3-by-7 array, as can be seen in Figure 9. The cameras had focal lengths of 3.6 mm and were placed at equal baseline distances of 21.1 mm from each other. The cameras can simultaneously capture the scene in still or video formats. To optimize the CII results, the cameras were aligned and calibrated [36]. Figure 10 shows an output example of this camera system, capturing a scene of toy objects.

We used 15 different scenes, where each scene provided 20 pairs of stereo images: 12 pairs with a varying horizontal baseline and 8 pairs with a vertical or diagonal baseline.

**Figure 11:**
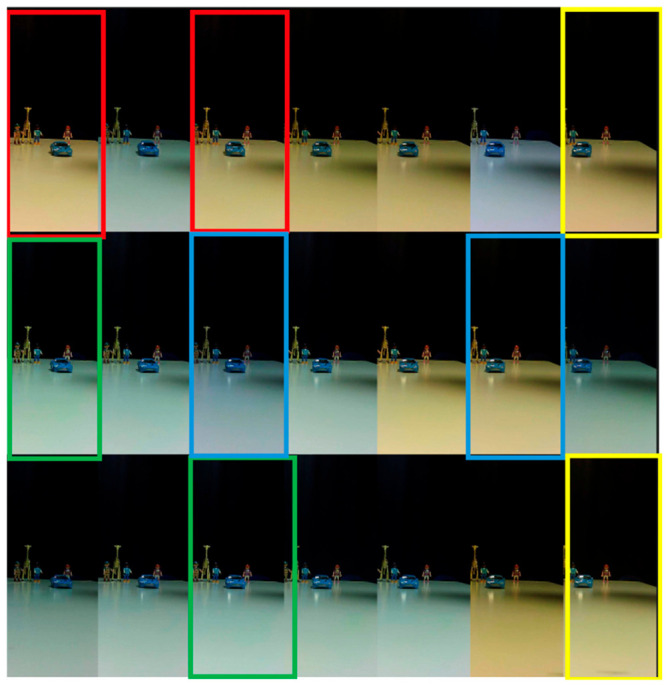
Examples of diverse stereo pairs within the array of elemental images, including a conventional horizontal alignment (red rectangles), a vertical alignment (yellow rectangles), a diagonal alignment (green rectangles), and a variety of baselines (blue rectangles and other combinations).

Usually, stereo matching models are trained using images captured by a stereo camera setup that has a fixed horizontal baseline. An example of a horizontal pairing can be seen in Figure 11, marked with red rectangles.

Exploiting the geometry of the input multi-view images, we generated other stereo pairing orientation types, as demonstrated by the yellow and green rectangles in Figure 11, illustrating vertical and diagonal orientations, respectively.

An additional advantage of the image array geometry is that this setup included multiple options for baselines (distances between cameras). An example can be seen in Figure 11, where a variety of baselines can be observed between the different cameras (rectangles) at the different orientations.

We implemented the stereo matching models [25,26,27] using Pytorch 2.4.0. First, we trained the models on Scene Flow dataset [23] in the same way as described in these papers. Then, we fine-tuned the models on our data. The models were trained for 300 epochs using the Adam optimizer ($\beta_{1}=0.9,\;\beta_{2}=0.999)$. The batch size was set to 4. The learning rate was set to 0.001 for 200 epochs and then 0.0001 for the remaining 100 epochs. Our data was divided into an 80/20 train/test split. Each scene consisted of 1–5 objects. The CII depth plane formation process (Equation (1)) used 3 [mm] depth intervals. The training process was carried out using an NVIDIA GeForce RTX 4070ti GPU. The computational complexities are similar to those of the chosen stereo matching models, since we train the same model architectures (just with different ground-truth data and loss function). The CII-based depth extraction is performed once to create the ground-truth data.

## 6. Results of Depth Estimation Accuracy

To evaluate the proposed method, we examined several published stereo matching models. Specifically, PSMNet [25], GWCNet [26], and AANet [27] were selected for testing.

Since our method is based on detected objects in the scene, only a portion of the depth map contains the most valid depth information. For this reason, we focused only on the object ROIs and used these areas for the loss calculation. The predicted depth that was compared to the ground-truth depth was calculated from the stereo matching models’ predicted disparity maps.

Since the loss is computed only at the object regions in the stereo model output, standard stereo evaluation metrics such as EPE or D1-all [25,27] cannot be applied since they assume evaluation over the entire image. Therefore, we used Mean Squared Error (MSE) as the loss function and report the results using the relative error rate.

**Figure 12:**
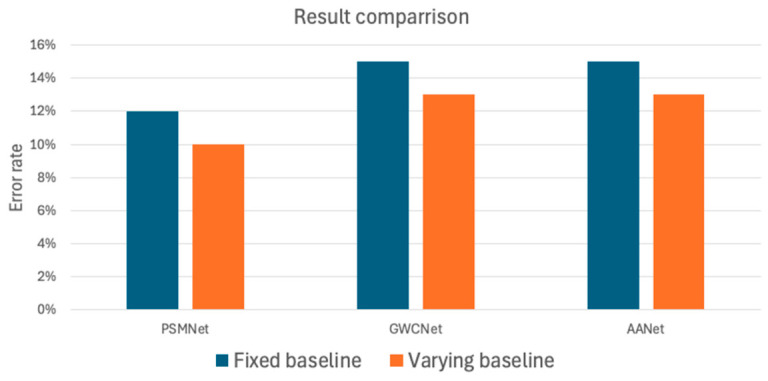
Comparison results of relative depth error based on PSMNet [24], GWCNet [25], and AANet [26] stereo matching model outputs using a fixed baseline (blue bars) and varying baselines and orientations (orange bars).

Figure 12 shows the experimental results. The chart compares the relative predicted depth errors of two training settings, a fixed baseline and a varying baseline, for three stereo matching models, PSMNet [25], GWCNet [26], and AANet [27]. The blue bars represent the fixed (conventional) baseline case, where stereo images were captured by cameras placed on the same horizontal axis with a fixed distance between them. These results show that all three models are able to learn depth information using object-level depth values instead of disparity maps.

The orange bars represent the varying-baseline case, where stereo images were captured using different camera distances and cameras that were not always aligned on the same horizontal axis. Importantly, the testing data included stereo image pairs with baselines that were not seen during training. The improved performance in this case shows that training with varying baselines and orientations enabled the models to generalize better to unseen camera configurations.

## 7. Conclusions

In this research, we explored how Computational Integral Imaging (CII) can be used to train stereo matching models. First, we presented an improved CII-based depth extraction method [11] by employing object masks instead of bounding boxes. An object segmentation mask excludes regions within its bounding box that belong to other objects or backgrounds in the scene. These regions are usually at different depths than the object and thus reduce the accuracy of the extracted object depth due to their less sharp appearance in the object’s depth plane. Therefore, with the modified approach, more accurate depths were produced, especially when objects were close to each other in the 2D image.

Next, we trained stereo matching models using the depth values produced by the improved CII-based method as the ground truth instead of traditional non-synthetic disparity maps often obtained using active imaging [6,7]. Since stereo matching models output full-image disparity maps while the CII-based method provides depth values for each object in the image, we used object detection and segmentation to link each object region in the disparity map to its estimated depth. YOLOv9 was used to locate objects, and SAM2 was used to define their exact regions.

The number of cameras and their arrangement in the camera array system can influence the diversity of the stereo pairs used in stereo matching training. It was previously shown [45] that a structure of at least 3 × 3 cameras is required to obtain a reliable object depth.

With this approach, the same modality, an RGB camera array, is used to obtain the ground-truth depth data and the spatial object ROIs in the image used for stereo matching. In this way, we avoid the need to associate depth data obtained from a different modality, such as LiDAR, with spatial locations in the RGB image.

The proposed method currently uses depths only at detected object regions in the image while ignoring background regions that exist in disparity maps. Hence, the stereo matching models were trained here only on regions containing detected objects. This means that the generated disparity maps do not fully represent the input images in the same way as the originally trained models. For this reason, common evaluation metrics such as End-Point Error (EPE) and D1-all are not suitable for evaluating the results in this case. While background regions can be considered less meaningful in various applications, potential future research may expand the usage of object-aware CII for other parts in the image beyond detected object regions. We used the segmentation model output to generate a mask for each detected object in a scene. However, current segmentation models such as SAM2 (Segment Anything Model 2) [43] can segment all the image regions and are not limited to predefined or classified objects. Future research may investigate the ability to estimate the depth of other/background segmented parts of the image (i.e., any segment-aware computational integral imaging). Some segments of the image may need additional validation and analysis.

Regarding the robustness of CII-based depth extraction to noise, in cases of mild texture, low contrast, or poorly illuminated objects, the gradient values may be very low, and therefore, the AGMR may be affected by random noise. However, our method is uniquely noise-resistant due to the strong summation operation in the computational integral imaging process that forms the depth planes (Equation (1)). In this process, at the correct depth-plane of a certain object, the sharp object gradients are summed coherently throughout all the elemental images, while random noises are summed incoherently, increasing the SNR. At the incorrect depths, both the noise and the object gradients are summed incoherently throughout the elemental images, keeping the SNR low. The robustness of our CII-based depth estimation general approach (a weaker and non-object-aware version of the method) was shown in a previous study [14].

Here, we present a proof of concept for training stereo matching models using CII-based depth data directly, without the need for modality adjustment. We have also shown that multi-view data provides an advantage because it contains diverse stereo pairs of the same scene, while most stereo systems use horizontal camera pairs. Adding vertical and diagonal pairs, as well as pairs with different baselines, improved the models’ ability to generalize. This is important because real-world stereo systems may have different baselines or slight alignment errors.

## Data Availability

The original contributions presented in this study are included in the article. Further inquiries can be directed to the corresponding author.
